# Clinical Experiences of Korean Medicine Treatment against Urinary Bladder Cancer in General Practice

**DOI:** 10.1155/2016/3759069

**Published:** 2016-04-12

**Authors:** Taeyeol Park, Sanghun Lee

**Affiliations:** ^1^Kyeongin Traditional Korean Medicine Clinic, 84-3 Dadae 2-dong, Saha-gu, Busan, Republic of Korea; ^2^Department of Medical Consilience, Graduate School, Dankook University, 152 Jukjeon-ro, Suji-gu, Yongin-si, Gyeonggi-do 448-701, Republic of Korea

## Abstract

Urinary bladder cancer (UBC) is one of the most common cancers, with 1 out of every 26 men and 1 out of every 80 women worldwide developing the disease during their lifetime. Moreover, it is a disease that predominantly affects the elderly and is becoming a major health problem as the elderly population continues to rapidly increase. In spite of the rapid development of medical science, the 5-year survival rate has remained around 75% since the 1990s, and the FDA has approved no new drugs for UBC over the last 10 years. In addition, most patients experience frequent recurrence and poor quality of life after diagnosis. Therefore, in order to solve unmet needs by alternative methods, we present our clinical cases of UBC where we observed outstanding results including regression and recurrence prevention exclusively through Traditional Korean Medicine such as (1) herbal therapy, (2) acupuncture, (3) pharmacopuncture and needle-embedding therapy, (4) moxibustion, and (5) cupping therapy. From our experience, it appears that multimodal strategies for synergistic efficiency are more effective than single Korean Medicine treatment. We hope this will encourage investigation of the efficacy of Korean Medicine treatment in clinical trials for UBC patients.

## 1. Introduction

Urinary bladder cancer (UBC) is a common disease with more than 12 million new cases annually worldwide, which ranks ninth in worldwide cancer incidence [1]. UBC occurs most commonly in the elderly: the median age at diagnosis is 69 years for men and 71 years for women in the USA [2]. It is therefore likely that it will become a greater health problem as the ageing population increases globally [3]. Up to now, surgical resection of the tumor has been the best treatment, but about 70% of patients experience subsequent recurrence, often in different locations from the initial tumor [4]. After repeated resections, the tumor usually becomes more aggressive, and the patients will finally be obliged to receive radical cystectomy. For the remainder of their life, they must endure suffering without their urinary bladder [5].

Therefore, the remission of UBC without surgical resection and the prolongation of the relapse period are goals in the treatment of UBC. From our own clinical experiences from general practice with Korean Medicine (KM), we suggest here that KM could be beneficial in achieving those goals. The state of UBC can be interpreted according to the categories of “溺血” (hematuria), “血淋” (blood stranguria), and “*癃閉*” (obstruction of urine flow), as written in the books of Traditional Asian Medicine [6, 7]. In China, clinical practice guidelines on various cancers including UBC were recently published for the first time based on the integration of western medicine and Traditional Chinese Medicine (TCM) [7]. The use of TCM without western medicine is only offered to support patients who fail chemotherapy treatment or are in a state too poor to receive western treatment. However, the Korean medical system is different from China in this respect in that Korea has completely dualized Korean Medicine and western medicine [8]. Therefore, the spectrum of KM is broader than that of TCM and could be a potential option for curative treatments, for example, in patients awaiting surgical resection. In this paper, we introduce the multimodal treatments of KM and several outstanding UBC cases.

## 2. Methods for Korean Medicine against Bladder Cancer (KMBC)

### 2.1. Herbal Therapy

The KM textbook, Donguibogam, says that urine stored in the bladder can only be excreted through the “氣” (Qi) transformation [6]. In the body, fluid circulation is made by Qi, which is written as “水” (Water), with Qi deemed to be the parent of Water (the son). “肺” (the Lungs) are the major organ involved in controlling “水道” (Waterways) such as the vessels that deliver urine to the bladder. As such, urination problems related to the bladder can be handled with Qi in the Lungs. The shortage of Qi incapacitates the flow of the Waterways and can be improved by boosting the Qi in the Lungs. The herbal remedy, “*生脈散*” (Saeng-Maek-San, SMS), at “Internal Bodily Elements part I” in Donguibogam has been suggested to improve the shortage of Qi in the Lungs [6]. Based on this background, SMS composed of Liriopis Tuber (tuber of* Liriope platyphylla*, Liliaceae), Ginseng Radix (root of* Panax ginseng*), and Schisandrae Fructus (fruit of* Schisandra chinensis*) was selected as the major herbal decoction and prescribed to UBC patients. Depending on the status of the patients, herbs such as Astragali Radix (root of* Astragalus membranaceus*) and* Oldenlandia diffusa* were added in order to improve energy levels and increase the anticancer effects. The herbal remedies, “猪*苓湯*” (Jeoryengtang) or “八正散” (Paljeongsan), were also administered in some patients to manage lower urinary tract symptoms. In the case of hematuria, herbs such as Rehmanniae Radix (root of* Rehmannia glutinosa*), node of Lotus rhizome, and Typhae Pollen were added. The decoction was prepared from a mixture of chopped crude herbs which were extracted twice in water at 100°C for 4 hours. The quality of the herbs was tested according to the Korea Food & Drug Administration (K-FDA). Oral administration of 100 mL decoction was prescribed three times a day.

### 2.2. Acupuncture

The acupuncture treatment is also based on an acupuncture theory in Donguibogam. By stimulating acupoints using a needle, it helps Qi circulate harmoniously in the body through balancing of “陰” (Yin) and “陽” (Yang). The skin was cleaned with alcohol before each insertion. Acupuncture needles (stainless steel, single-use, sterile, and disposable, 0.25 × 30 mm length; DongBang Acupuncture, Inc., Korea) were inserted perpendicularly. The major acupoints are LI04, LR03, KI03, SP09, CV3, CV4, ST29, BL22, BL23, BL32, BL40, and BL52, the location of each based on the WHO standards. The acupoints BL65 and BL67 were also added in the case of urinary symptoms such as frequent and painful urination, and BL17, SP06, and SP10 were added in cases of hematuria dependent on the lower urinary tract symptoms. The acupuncture stimulation should make patients experience a dull or achy feeling known as the “得氣” (De Qi) sensation. The CV3 and CV4 locations anatomically adjacent to the bladder are given particular attention as a precaution to ensure no bladder puncture. It is recommended that the procedure for these points is undertaken after urination. The acupuncture treatments should be administered for at least 3 sessions per week lasting 20–25 minutes.

### 2.3. Pharmacopuncture and Needle-Embedding Therapy

Pharmacopuncture is a treatment injecting herbal medicine extracts into acupoints in order to enhance the mechanical and chemical effect of acupuncture and herbal medicine. “Herbs part VII” in Donguibogam says that* Nidus Vespae* is nontoxic and cures urination difficulties and stubborn abscesses, the latter through external use [6]. The* Nidus Vespae* pharmacopuncture solution was obtained by the guideline of the pharmacopuncture preparation at an extramural facility meeting Korean Good Manufacturing Practice (K-GMP) standards [9]. The final solution was stored at 4°C. The* Nidus Vespae* pharmacopuncture treatment was conducted using 30-gauge sterile disposable syringes (BD Ultra-Fine*™* Needle, USA). After sterile skin preparation, the selected acupoints mentioned above were stimulated with a perpendicular, subcutaneous injection at a depth of 0.5 to 1.0 cm with 0.1 to 0.2 mL of the solution. The treatment can be given on twice-a-week basis for six months and a maintenance treatment may be given weekly. Embedding therapy is also referred to as medicinal thread inserting therapy in order to elongate the duration of stimulation on the acupoints. The harmless catgut threads were used (Miracu*™*, DongBang Acupuncture, Inc., Korea). The needle-embedding therapy was also performed on the several acupoints mentioned above depending on the status of the patients, which followed the general protocols [10]. It can be given once at two-week intervals for six sessions.

### 2.4. Moxibustion

Moxibustion has also the intention of stimulating Qi circulation by heating the acupoints through the burning of moxa made from dried mugwort (*Artemisia argyi*). The use of moxibustion can be divided into two methods, direct moxibustion and indirect moxibustion, depending, respectively, on whether moxa is in direct contact with the skin or not [11]. Direct moxibustion is seen to be more effective than the indirect method, but it causes skin burns. Patients with diabetes or edema should receive moxibustion only with careful monitoring by a KM doctor. Moxibustion points include the seven local acupoints of CV2, CV3, CV4, CV12, BL13, BL23, and BL28 affecting the anatomical bladder and the bladder meridian. The treatment should be conducted after sterilizing the skin surface at the acupoints and administered during at least 3 sessions per week. A dressing with povidone-iodine-containing local therapeutics can help to restore skin burns.

### 2.5. Cupping Therapy

The cupping therapy also helps Qi circulation through local suction created on the skin. It can be divided into two methods, wet-cupping and dry-cupping, depending, respectively, on whether a small quantity of blood was drawn out by vacuum or not [12]. The wet-cupping therapy is seen to be more effective than dry-cupping, but there is a risk of infection due to skin injuries. Correct sterilization is essential before the procedure is carried out, and only disposable cups must be used. Treatment points are located bilaterally at BL23, BL27, BL28, ST28, and ST29. We used 40 cc disposable cups (DongBang Acupuncture, Inc., Korea) and disposable caps for the autolancets. One or two cupping therapy sessions per week were recommended.

## 3. UBC Cases

### 3.1. UBC Regression Waiting for Surgical Resection

**Figure 1:**
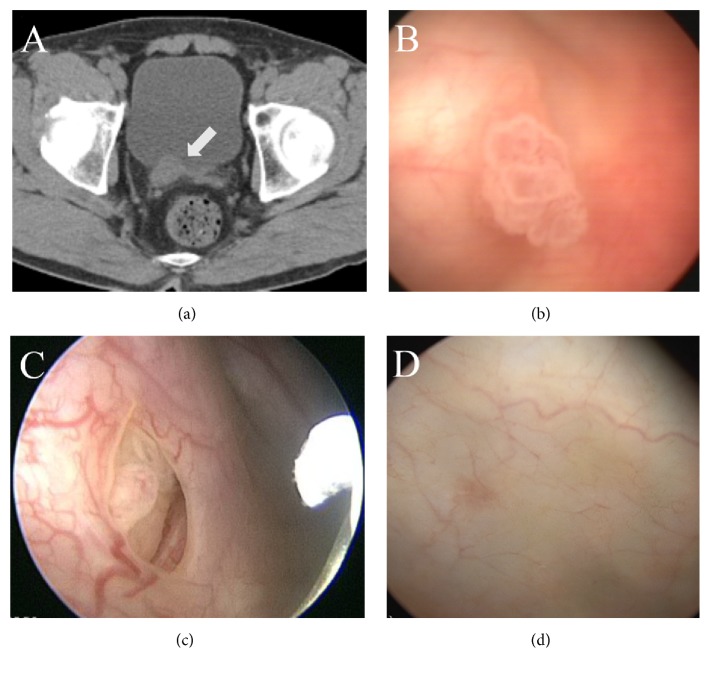
From the upper left, CT scans (a) showed the papillary urothelial carcinoma at the distal right ureter and initial cystoscopy (b) found another mass in the urinary bladder (May 2015). After treating solely with the Korean Medicine treatment, follow-up cystoscopy (c and d) in June and August 2015, respectively, demonstrated its complete remission.

 The cases are as follows:

1. A 59-year-old Asian man who presented with hematuria was identified with two masses at the distal right ureter and bladder by CT scans and cystoscopy in May 2015 (Figures 1(a) and 1(b)). Surgical resection was planned for one month later. Whilst waiting for the operation, he received the KMBC treatment for 38 days. Ureteroneocystostomy was performed in June 2015 and papillary urothelial carcinoma was pathologically confirmed with invasion into subepithelial connective tissue (pT1) and high grade (Gr 2). However, the other mass in the bladder, which had been scheduled to be removed transurethrally, could not be found (Figure 1(c)). Any UBC has not been found up to the last follow-up (October 2015).
2. A 37-year-old Asian man was diagnosed with papillary urothelial carcinoma in June 2013. Multiple masses occurred at the anterior and posterior wall in his bladder in January 2015. Transurethral resection of the bladder tumor (TURBT) was recommended, but he delayed it because he was afraid of the frequent recurrence of UBC in spite of resection. The KMBC treatment was started from January 2015. After the KMBC treatment alone, cystoscopy was carried out in June 2015, revealing that the smaller masses had disappeared and the bigger masses had decreased as compared with the previous 5 months.

### 3.2. Frequently Recurrent pT2NxM0 Stage

A 71-year-old Asian woman with a past medical history of hypertension and diabetes who presented with urinary frequency and nocturia was diagnosed with urothelial carcinoma with pT1 in May 2013. TURBT was performed, followed by Bacillus Calmette-Guérin (BCG) vaccine treatment. Thereafter, she received TURBT three times against the recurrent UBC in August 2013, November 2013, and March 2014. Finally, the pathological T2 stage and high grade (Gr 3) were confirmed with invasion into subepithelial connective tissue and muscularis propria, micropapillary component (50%), and lymphovascular invasion was present. Cystectomy was therefore recommended for her due to disease progression. After considering the quality of life she would have without a bladder for the rest of her life, she decided to start KMBC treatment from March 2014. After the KMBC treatment alone, cystoscopy was carried out every 3 months, and no recurrent UBC was found up to the last follow-up (September 2015). Her period of disease-free survival (DFS) is over 18 months, which is exceptionally long when compared with her past frequency of recurrence every 3 months and muscle invasion.

### 3.3. Adenocarcinoma with pT1N0M0 Stage

A 58-year-old Asian woman who had been working as a painter in a shipbuilding yard for several years presented with hematuria. She received TURBT and was diagnosed with adenocarcinoma with pT1N0M0 in March 2011. Unfortunately, her UBC recurred 4 months later and TURBT was performed revealing the same diagnosis, adenocarcinoma with pT1 stage. In order to prevent tumor recurrence, she decided to start the KMBC treatments from October 2011. After the KMBC treatment alone, cystoscopy was carried out every 3 months, and no recurrent UBC has been found up to the last follow-up (November 2014). Her DFS is over 3 years, which is very long considering that her UBC is an adenocarcinoma that recurred in 4 months with poor prognosis compared with urothelial carcinoma.

### 3.4. Frequently Recurrent pTaN0M0 Stage

 The cases are as follows:

1. A 53-year-old Asian man with past nonspecific medical history presented with urinary frequency, urgency, and nocturia. He received TURBT and was diagnosed with papillary urothelial carcinoma with pTa and low grade (Gr 1) in January 2010. This was followed with BCG vaccine treatment. However, he received TURBT eight times against the recurrence of his UBC up until October 2014. Eventually, the pathological grade increased to Gr 2 and his UBC relapsed three times during the last 6 months. After growing tired of the frequently recurrent tumor, he decided to start the KMBC treatment from October 2014. After the KMBC treatment alone, cystoscopy was carried out every 3 months and no recurrent UBC has been found up to the last follow-up (September 2015). His DFS is around 12 months, which is long compared with his past frequent recurrence in 6-month intervals. Additionally, other urinary symptoms such as painful and frequent urination were improved after KMBC treatment.
2. A 39-year-old Asian man with past nonspecific medical history presented with hematuria and after receiving TURBT was diagnosed with papillary urothelial carcinoma with pTa and Gr 2 in April 2013. Thereafter, he received TURBT twice against recurrent UBC in January 2014 and May 2014. His disease progressed with the number (4 *←* 1) and the region of UBC broadened and expanded to the whole bladder. Due to fear of a frequently recurrent tumor, he decided to start the KMBC treatment from June 2014. After the KMBC treatment only, cystoscopy was carried out every 3 months and did not reveal any recurrent UBC up to the last follow-up (October 2015). His DFS is around 17 months, which is long compared with his past frequent recurrence and disease progression.

## 4. Discussions

Cancer is a very complex disease, characterized by sustained proliferative signaling, evasion of growth suppressors, resistance to cell death, replicative immortality, induction of angiogenesis, and the activation of invasion and metastasis [13, 14]. Besides the cancer cells themselves, aspects of the tumor microenvironment such as stroma or immune cells have also been found to play a key role in tumorigenesis [14]. For these reasons, the western treatment strategy of focusing and targeting only the cancer cells could have lots of limitations [15]. Therefore, a multitarget therapeutic approach is a relevant strategy for addressing the biological complexity of cancer development and one that could possibly be realized with botanicals through synergistic interaction or multifactorial effects between various compounds present in herbal extracts [16, 17]. Recently, a complex herbal formula from KM designed to holistically modulate a person's physiological/pathological networks could act as a blueprint for a new generation of medicine based on integrated network-based medicine [18].

In the KMBC treatments, the main herbal remedy is SMS (*生脈散*), literally meaning to encourage (生) the energy (脈) in our body, which is interpreted as the ability to enhance immune function. Scientifically, it has been proven to increase tumor necrosis factor- (TNF-) *α* and interleukin- (IL-) 6 levels with immunological activity enhancement in thymocytes and splenocytes as well as boost the phagocytic activity of macrophages [19, 20]. Several studies on the direct effect against UBC have suggested that a homogeneous polysaccharide from* Panax ginseng* displayed potent antiproliferative and antimetastatic activities in human bladder T24 cells, and Rg3 ginsenoside inhibited the proliferation of EJ (human bladder transitional cell carcinoma cells) by inducing apoptosis [21, 22]. Additionally, treatment with* Liriope platyphylla* significantly inhibited proliferation of MCF-7 (breast carcinoma cells) and Huh-7 (hepatic carcinoma cells) by inducing apoptosis and autophagy pathways [23]. DT-13, a saponin monomer from* Liriope platyphylla*, showed antiangiogenic effects mediated by reductions in vascular endothelial growth factor (VEGF), C-C chemokine receptor type 5 (CCR5), and hypoxia-inducible factor 1*α* (HIF-1*α*) [24]. Schizandrin B, one of the main dibenzocyclooctadiene lignans present in Schisandrae Fructus, was also shown to have an anticancer effect by blocking the invasion and migration of lung adenocarcinoma A549 cells through downregulation of expression of HIF-1, VEGF, and matrix metalloproteinase (MMP) [25]. A crude extract from* Schisandra chinensis* has a remarkable reversal effect on multidrug resistance in cancer cells by inhibiting the function and expression of P-glycoprotein and protein kinase C [26].

Externally applied onto the acupoints,* Nidus Vespae* is experimentally proven to increase TNF-*α* and IL-6 secretion of monocytes and the IgG production of B cells and promote the phagocytosis of tumor cells by monocytes, effects similar to the SMS herbal remedy [27]. Propolis (bee glue), a bee-metabolized resinous mixture of* Nidus Vespae*, has been used as a healing agent since ancient times because of various biological effects, which were validated to be antimicrobial, antioxidant, anti-inflammatory, antidiabetic, dermatoprotective, antiallergic, laxative, immunomodulatory, and anticancer [28, 29]. Recently, it has been suggested as a potential source of adjuvant drugs for bladder cancer treatment because of the cytotoxicity in human superficial bladder cancer cells, antiangiogenic effects in rat bladder cancer, and chemopreventive effects against bladder chemical carcinogenesis [30–32]. Therefore, our outstanding clinical cases showing UBC regression or recurrence prevention are successful examples of a multitarget therapeutic strategy, both internally and externally, which exhibits the synergistic efficiency of multiextract combinations used presently in KM.

In Donguibogam, “積聚” (Jeok-Chi) was described as a tangible disease with hardness, which is quite similar to tumors [6]. It was seen to mostly develop from the stagnation of Qi, which is interpreted as localized hypoxic conditions with diminished local blood circulation that promotes inflammation and tumor growth [33, 34]. Qi stagnation has also been recorded as being improved by the stimulation of acupoints on the meridian in the body, because the meridian system in Traditional Asian Medicine is a path of Qi [6, 15]. Traditionally, acupuncture, moxibustion, and cupping therapy have been used to stimulate the acupoints on the meridian for Qi flow.

With the progress of research, acupuncture has become recognized and practiced as adjuvant treatment for cancer patients in treating various symptoms in western countries because of its modulatory effects on the nervous, endocrine, and immune systems [35, 36]. In addition to this, it can provide a beneficial effect in anticancer treatment by promoting IL-2, T cell subtypes, and natural killer cells in lung cancer patients [37, 38]. Recently, the anticancer mechanism in acupuncture has been explained to be a result of purinergic signaling involved in diseases of the lower urinary tract including UBC [39]. Treatment of bladder cancer with adenosine 5′-triphosphate (ATP) was confirmed to be effective via P2X5 and P2X7 ion channel receptors in animal models and human cell lines, and it also improved the systemic symptoms associated with advanced malignancy [40]. In light of this, the mechanical deformation of the acupoints on the skin by acupuncture, moxibustion, and cupping therapy in the KMBC treatments induces the release of large amounts of ATP from keratinocytes, fibroblasts, and other cell types in skin, which is beneficial for the inhibition of UBC as well as the symptoms of the lower urinary tract [41, 42].

The acupoints selected in KMBC treatments are commonly known to affect the micturition center and parasympathetic innervation to the urinary system [43, 44]. These places around the navel, sacrum, and legs are organized segmentally with the bladder, which is innervated peripherally by the sympathetic nerves originating at T11-L2, as well as the parasympathetic and somatic nerves originating at S2–S4. Several clinical studies have verified that stimulation on these acupoints alleviates pain, urinary symptoms, and quality of life in patients with an overactive bladder or chronic prostatitis/chronic pelvic pain syndrome (category IIIB) [45, 46]. In our UBC cases, these improvements were also observed, though urinary complaints such as frequency, urgency, and nocturia could not be evaluated by an official symptom assessment tool.

In conclusion, our clinical experiences in general practice suggest that multimodal strategies based on KM could be a safe and effective treatment in managing UBC. They seem to be a good alternative in preventing the recurrence of UBC after surgical resection given that approximately 70% of UBC patients go into relapse despite adjuvant BCG or chemotherapy. In particular, the first two cases suggest that KMBC treatment can be used as a neoadjuvant treatment or an alternative in inoperable status. Large, well-designed randomized clinical trials are necessary for this conclusion because the clinical evidence from our study is insufficient. However, it should be considered that multimodal KM treatments in general practice make it difficult to be standardized and blinded in clinical trials.
